# SAA1 gene variants and childhood obesity in China

**DOI:** 10.1186/1476-511X-12-161

**Published:** 2013-10-30

**Authors:** Xiao Zhang, Qi-Zhu Tang, Ai-Ying Wan, Hai-Ju Zhang, Li Wei

**Affiliations:** 1 Department of Pediatric, RenMin Hospital of Wuhan University, Jie Fang Road, Wuchang district, Wuhan 430060, China

**Keywords:** Genetic polymorphisms, Serum amyloid A, Obesity, Childhood

## Abstract

**Background:**

Obesity increases the risk for insulin resistance and metabolic syndrome in both adults and children. SAA is a member of apolipoprotein and plays an important role in maintaining glucose and lipid homeostasis. The purpose of this study was to assess SAA1 allelic variants with obesity in young school-age children.

**Methods:**

A total of 520 consecutive children ages 5–15 years were recruited. Children were divided based on BMI z score into Obese (OB; BMI z score ≥1.65; n = 253) and non-obese (NOB; n = 267). Four SNPs of the human SAA1 gene (rs12218, rs4638289, rs7131332 and rs11603089) were genotyped by use of polymerase chain reaction – restriction fragment length polymorphism (PCR-RFLP) method.

**Results:**

Compared to NOB, circulating SAA levels were increased in OB, as were LDL-C, TG and TC concentration. Obese children showed increased frequency of rs12218 and rs4638289 polymorphism compared to control children. There were no differences between OB and NOB for the other 2 polymorphisms. Only the rs4638289 polymorphism showed significant contributions to higher SAA plasma levels.

**Conclusions:**

SAA1 genetic polymorphism was associated with obesity in Chinese children.

## 

Childhood obesity is a serious public health problem that has reached epidemic proportions all over the world [1,2]. In children, the presence of obesity has been associated with increased levels of high sensitivity CRP (hsCRP) [3], as well as other inflammatory mediators [4-8], all of which promote the development of endothelial and metabolic dysfunction [9-12]. Serum amyloid A (SAA) is not only a sensitive acute phase proteins in plasma, but also an apolipoprotein that can replace apolipoprotein A1 (apoA1) as the major apolipoprotein of HDL-C, particularly during the acute phase response [13]. Several studies suggested that SAA is associated with obesity in adult [14]. And a meta-analysis found a strong association between body mass index and SAA levels [15]. Previous studies have demonstrated that SAA1 genetic polymorphism can change the plasma SAA levels [16]. Therefore, the genetic polymorphisms in SAA1 may be associated with obesity. Furthermore, several studies have reported that rs12218 in SAA1 gene was associated with carotid atherosclerosis [17], HDL-C concentration [18,19] and peripheral arterial disease [20]. However, the relationship between SAA gene polymorphism and obesity remains unknown.

In the present study, we aimed to investigate the relation between SAA1 genetic polymorphism and obesity in Chinese children.

## Results and discussion

A total of 253 OB children and 267 age-, gender- and ethnicity-matched NOB children were recruited during January to July 2012. The demographic characteristics of this cohort are shown in Table 1. As anticipated, OB children had higher LDL-C, TC and TG levels compared to NOB children (Table 1). OB children also had significantly higher levels of SAA than NOB (Table 1).

**Table 1:** Characteristics of the participants

**Groups**	**N**	**Age (years)**	**BMI Kg/m**^**2**^	**SBP (mmol/L)**	**DBP (mmol/L)**	**GLU (mmol/L)**	**TG (mmol/L)**	**TC (mmol/L)**	**HDL-C (mmol/L)**	**LDL-C (mmol/L)**
OB group	253	13.2 ± 0.9	21.1 ± 2.6	99.5 ± 12.6	61.0 ± 7.9	4.43 ± 0.47	0.61 ± 0.28	2.8 ± 0.7	0.87 ± 0.23	1.39 ± 0.49
NOB group	267	13.4 ± 1.0	17.1 ± 1.4	97.7 ± 12.7	61.4 ± 7.0	4.35 ± 0.48	0.52 ± 0.17	2.6 ± 0.6	0.88 ± 0.22	1.25 ± 0.36
*P*		0.102	<0.001	0.113	0.506	0.055	<0.001	0.001	0.734	<0.001

The frequency of each of the SAA polymorphisms is shown in Table 2 for OB and NOB children. Obese children showed increased frequency of rs12218 and rs4638289 polymorphism compared to control children. There were no differences between OB and NOB for the other 2 polymorphisms. Only the rs4638289 polymorphism showed significant contributions to higher SAA plasma levels (Table 3).

**Table 2:** Distributions of SAA1 genotypes (OB = 253, NOB = 267)

**SNPs**	**Allels (1/2)**	**Groups**	**Genotypes (n, %)**			***P *** **value**
			**1/1**	**1/2**	**2/2**	
rs11603089	A/G	NOB group	192 (0.72)	70 (0.26)	5 (0.02)	0.302
		OB group	172 (0.68)	71 (0.28)	10 (0.04)	
rs4638289	A/T	NOB group	107 (0.40)	134 (0.50)	26 (0.10)	0.001
		OB group	84 (0.33)	114 (0.44)	55 (0.23)	
rs12218	C/T	NOB group	152 (0.57)	102 (0.38)	13 (0.05)	0.014
		OB group	129 (0.51)	94 (0.37)	30 (0.12)	
rs7131332	A/G	NOB group	67 (0.25)	160 (0.60)	40 (0.15)	0.897
		OB group	65 (0.24)	147 (0.58)	41 (0.18)	

**Table 3:** SAA concentration between each genotypes ( M + SD; ug/mL)

**Group**	**n**	**rs11603089**			**rs4638289**			**rs12218**			**rs7131332**		
		**AA**	**AG**	**GG**	**AA**	**AT**	**TT**	**CC**	**CT**	**TT**	**AA**	**AG**	**GG**
OB group	253	71.16 ± 23.22*	68.03 ± 22.11*	70.13 ± 24.13*	62.62 ± 23.22*	78.26 ± 24.32*	88.24 ± 30.13*^△^	73.15 ± 25.23*	70.03 ± 23.44*	69.18 ± 25.14*	68.64 ± 24.91*	73.65 ± 25.86*	70.60 ± 23.12*
NOB group	267	51.22 ± 21.13	52.02 ± 22.11	53.12 ± 24.87	45.54 ± 20.53	59.12 ± 24.86	62.44 ± 24.34^△^	55.33 ± 24.31	50.34 ± 26.15	55.29 ± 22.96	52.71 ± 21.43	50.1 ± 24.49	53.71 ± 26.05

Note: compared to NOB group, *P < 0.05; Compared to AA genotype of rs4638289, ^△^P < 0.05.

In the present study, we found that both rs12218 and rs4638289 polymorphism in the SAA1 gene were associated with OB in Chinese Children. And the SAA plasma levels are significantly higher in obese children. Furthermore, there was significant difference in SAA plasma levels between each genotype of rs4638289 in SAA1 gene.

As described previously [18,20], SAA1 encodes one important inflammation factor-SAA.. Recently, Xie et al. reported that rs12218 polymorphism in SAA1 gene was associated with IMT [17], HDL-C [18,19], Ankle-brachial index (ABI) [20], and plasma uric acid levels [18] which was related to cardiovascular disease in adults. And Zhang et al. reported SAA1 gene polymorphism was associated with cerebral infarction [21]. Xu et al. also reported SAA1 gene polymorphism was associated with lipid levels [19]. However, the relationship between SAA gene polymorphism and obesity in Children remains unclear.

Inflammation is important in the pathogenesis of atherosclerosis and obesity [15]. In 2000, Yamada et al. [22] reported that the SAA1 genetic polymorphism influences the plasma concentration of SAA. In the present study, we performed a case–control study to observe the relationship between SAA1 genetic polymorphism and obesity. We found rs12218 CC genotype and rs4638289 TT genotype are very common in the obesity patients than that in the control subjects. Furthermore, we also find the rs4638289 was associated with SAA level, but the rs12218 was not found to be associated with SAA level in the present study. An elevated level of SAA causes amyloidosis and is a risk factor for atherosclerosis and its clinical complications, type 2 diabetes, as well as various malignancies. The previous study [23] described the first genome-wide association study on baseline SAA concentrations. In a meta-analysis of four genome-wide scans totalling 4,212 participants of European descent, the authors identified two novel genetic susceptibility regions on chromosomes 11 and 1 to be associated with baseline SAA concentrations. The chromosome 11 region contains the serum amyloid A1 gene and the adjacent genes and explains a high percentage of the total estimated heritability. In their study, the also found rs463889 was associated with SAA concentration. Our result was in line with their report. However, the mechanism which may link SAA1 genetic polymorphism to obesity is still unknown. The change of plasma concentration of SAA resulting from the genetic polymorphism of SAA1 may be a possible mechanism which merits further investigation.

In conclusion, SAA1 genetic polymorphism was associated with obesity in Chinese children.

### Subjects and methods

#### Subjects

The study was approved by the Wuhan University Human Research Committee, and informed consent was obtained from the legal caregiver of each participant. Consecutive children between the ages of 5 to 15 years attending public schools in Wuhan city were invited to participate in the study, after they underwent a school based health screening, which included height and weight measurements. Based on such screening, children were identified when their BMI z score was ≥ 1.65 (OB) and age-, gender-, ethnicity-, and area-of residence-matched children with BMI z scores <1.65 (NOB) were then identified and recruited to serve as controls. Of note, all children were otherwise healthy, and were representative of the demographic characteristics of the general population of the city of Wuhan. Children were excluded if they had known diabetes or pre-diabetes, any defined genetic abnormality or underlying systemic disease including hypertension, or if they were within a month from any acute infectious process.

#### Genotyping

The selection and genotyping of SNPs was performed according to Xie et al.’s protocol [20]. Briefly, we analyzed four tagging SNPs (rs12218, rs4638289, rs7131332 and rs11603089) in the present study. Genomic DNA was extracted from the peripheral blood leukocytes using a DNA extraction Kit (Beijing Bioteke Co. Ltd). Genotyping was confirmed by polymerase chain reaction (PCR) – restriction fragment length polymorphism (RFLP) analysis. The primer pair sequences, annealing temperatures and restriction enzymes for these four SNPs were described in the previous study [18-20].

#### Anthropometry

To verify the school-health screening initial reports, children were weighed in a calibrated scale to the nearest 0.1 kg and height (to 0.1 cm) was measured with a stadiometer (Holtain, Crymych, UK). Body mass index (BMI) was calculated and BMI z-score was computed using CDC 2000 growth standards http://www.cdc.gov/growthcharts and online software http://www.cdc.gov/epiinfo. A BMI z score ≥ 1.65 was considered as fulfilling the criteria for obesity.

#### Blood based assays

Blood samples were drawn by venipuncture in the morning after an overnight fast. Blood samples were immediately centrifuged and plasma was frozen at -80°C until assay. Serum lipids including total cholesterol, high-density lipoprotein (HDL) cholesterol, calculated low-density lipoprotein cholesterol (LDL), and triglycerides (TG) were assessed using Flex Reagent Cartridges (Dade Behring). Plasma SAA level was measured using enzyme-linked immunosorbent assay (ELISA kit, Beijing Nothern Biotechnology Institute, Beijing, China).

#### Statistical analyses

Analyses were carried out using SPSS version 17.0 (SPSS, Chicago, IL, USA). The Hardy–Weinberg equilibrium was assessed by chi-square analyses. The differences in the distribution of genotypes between OB and NOB were analyzed using the chi-square test. General linear model (GLM) analysis was performed to test for associations between SNP genotypes and BMI after adjusting for confounding variables. Normality was assessed by plotting the residuals. Correction for multiple testing was applied for the number of individual risk factors per genotype to achieve a probability value for significance to be assumed before analysis was undertaken.

## Abbreviations

SAA: Serum amyloid A; OB: Obesity; TG: Triglycerides; TC: Total cholesterol; HDL-C: High-density lipoprotein; LDL-C: Low-density lipoprotein.

## Competing interests

The authors declare that they have no competing interests.

## Authors’ contributions

XZ and QZT carried out the molecular genetic studies and drafted the manuscript. AYW and HJZ carried out the genotyping. LW, XZ and QZT participated in the design of the study and performed the statistical analysis. XZ and HJZ conceived of the study, and participated in its design and coordination and helped to draft the manuscript. All authors read and approved the final manuscript.
